# An eHealth Program for Insomnia in Children With Neurodevelopmental Disorders (Better Nights, Better Days): Protocol for an Economic Evaluation of a Randomized Controlled Trial

**DOI:** 10.2196/46735

**Published:** 2023-09-12

**Authors:** Xiao Yang Jia, Pantelis Andreou, Cary Brown, Evelyn Constantin, Roger Godbout, Ana Hanlon-Dearman, Osman Ipsiroglu, Graham Reid, Sarah Shea, Isabel M Smith, Jennifer D Zwicker, Shelly K Weiss, Penny Corkum

**Affiliations:** 1 The School of Public Policy University of Calgary Calgary, AB Canada; 2 Department of Community Health and Epidemiology Dalhousie University Halifax, NS Canada; 3 Faculty of Rehabilitation Medicine - Occupational Therapy University of Alberta Edmonton, AB Canada; 4 Department of Pediatrics McGill University Montreal, QC Canada; 5 Department of Psychiatry and Addiction Université de Montréal Montreal, QC Canada; 6 Department of Pediatrics and Child Health University of Manitoba Winnipeg, MB Canada; 7 Department of Pediatrics University of British Columbia Vancouver, BC Canada; 8 Departments of Psychology The University of Western Ontario London, ON Canada; 9 Department of Family Medicine Schulich School of Medicine and Dentistry The University of Western Ontario London, ON Canada; 10 Department of Paediatrics Schulich School of Medicine and Dentistry The University of Western Ontario London, ON Canada; 11 Children’s Health Research Institute London, ON Canada; 12 Department of Pediatrics Dalhousie University Halifax, NS Canada; 13 Department of Pediatrics Hospital for Sick Children Toronto, ON Canada; 14 Department of Psychology and Neuroscience Dalhousie University Halifax, NS Canada

**Keywords:** eHealth intervention, pediatric insomnia, neurodevelopmental disorders, attention-deficit/hyperactivity disorder, autism spectrum disorder, cerebral palsy, fetal alcohol spectrum disorder, economic evaluation, cost-effectiveness

## Abstract

**Background:**

Children with neurodevelopmental disorders have a high risk of sleep disturbances, with insomnia being the most common sleep disorder (ie, chronic and frequent difficulties with going and staying asleep). Insomnia adversely affects the well-being of these children and their caregivers. Pediatric sleep experts recommend behavioral interventions as the first-line treatment option for children. Better Nights, Better Days for Children with Neurodevelopmental Disorders (BNBD-NDD) is a 5-session eHealth behavioral intervention delivered to parents to improve outcomes (eg, Pediatric Quality of Life Inventory [PedsQL]) for their children (ages 4-12 years) with insomnia and who have a diagnosis of mild to moderate attention-deficit/hyperactivity disorder, autism spectrum disorder, cerebral palsy, or fetal alcohol spectrum disorder. If cost-effective, BNBD-NDD can be a scalable intervention that provides value to an underserved population.

**Objective:**

This protocol outlines an economic evaluation conducted alongside the BNBD-NDD randomized controlled trial (RCT) that aims to assess its costs, efficacy, and cost-effectiveness compared to usual care.

**Methods:**

The BNBD-NDD RCT evaluates the impacts of the intervention on children’s sleep and quality of life, as well as parents’ daytime functioning and psychosocial health. Parent participants were randomized to the BNBD-NDD treatment or to usual care. The economic evaluation assesses outcomes at baseline and 8 months later, which include the PedsQL as the primary measure. Quality of life outcomes facilitate the comparison of competing interventions across different populations and medical conditions. Cost items include the BNBD-NDD intervention and parent-reported usage of private and publicly funded resources for their children’s insomnia. The economic evaluation involves a reference case cost-effectiveness analysis to examine the incremental cost of BNBD-NDD per units gained in the PedsQL from the family payer perspective and a cost-consequence analysis from a societal perspective. These analyses will be conducted over an 8-month time horizon.

**Results:**

Research funding was obtained from the Kids Brain Health Network in 2015. Ethics were approved by the IWK Health Research Ethics Board and the University of Calgary Conjoint Health Research Ethics Board in January 2019 and June 2022, respectively. The BNBD-NDD RCT data collection commenced in June 2019 and ended in April 2022. The RCT data are currently being analyzed, and data relevant to the economic analysis will be analyzed concurrently.

**Conclusions:**

To our knowledge, this will be the first economic evaluation of an eHealth intervention for insomnia in children with neurodevelopmental disorders. This evaluation’s findings can inform users and stakeholders regarding the costs and benefits of BNBD-NDD.

**Trial Registration:**

ClinicalTrial.gov NCT02694003; https://clinicaltrials.gov/study/NCT02694003

**International Registered Report Identifier (IRRID):**

DERR1-10.2196/46735

## Introduction

### Background

Neurodevelopmental disorders (NDDs), estimated to affect 5%-9% of children [1,2], are a heterogeneous group of chronic conditions that affect the central nervous system. Children with NDDs are a vulnerable population that experiences significant health disparities compared to typically developing (TD) children, including high rates of sleep difficulties. In children with NDDs, sleep difficulties contribute to morbidity and impair cognition, emotional regulation, and neurobehavioral functioning [3]. Prevalence rates of sleep disturbances have exceeded 90% in children with certain NDDs, with insomnia being the most frequently cited sleep disorder [4]. Sleep disturbances can have a negative impact on children’s quality of life (QoL) and exacerbate stress in their caregivers [3].

Despite limited safety and efficacy data in children, pharmacotherapies, both over-the-counter and prescription, are often given to treat insomnia in this population [5]. However, pediatric sleep experts recommend that psychosocial interventions be implemented first [6]. Specifically, the intervention would start with psychoeducation and healthy sleep practices, and be followed by psychological interventions such as bedtime fading or modified extinction [7-9]. Then, if psychosocial interventions are ineffective or unfeasible, medications become an option [7-9].

The existing literature has demonstrated the efficacy of treating insomnia in TD children with psychosocial interventions [10,11]. While there is less research on children with NDDs, the available evidence also demonstrates the effectiveness of these interventions. A review of nonpharmacological interventions for sleep problems in youths with chronic health conditions concluded that the effectiveness of psychosocial interventions was not exclusive to specific health conditions but had comparable effects across children with a variety of mental and physical health conditions, including NDDs [12]. Psychosocial interventions for pediatric insomnia have also been shown to improve parents’ sleep and increase their feelings of competence and control [13,14].

Existing psychosocial interventions are typically delivered in conventional settings (eg, hospital clinics, outpatient professionals’ offices), consisting of weekly training sessions for parents [15-18] and several visits with health care providers [19,20]. Hence, time constraints and required travel expenses to health facilities may be access barriers for families. Reaching families in rural or remote communities and those who are financially constrained or lack insurance coverage for private psychological and other necessary clinical services are significant challenges in the delivery of behavioral interventions [21].

### Economic Evaluation

Economic evaluations can help guide policy making and resource allocation decisions. Little is known about the cost-effectiveness of eHealth (internet-based in this case) behavioral interventions for sleep problems in children. Many parents are interested in internet-based interventions for their children’s sleep problems [22]. An internet-delivered treatment program may be an effective tool to address and overcome barriers to accessing pediatric insomnia care [22,23]. The cost-effectiveness of internet-based sleep interventions for children in comparison to usual care has not been determined for children with NDDs [22].

The value for money of internet-based interventions can be assessed using cost-effectiveness analysis (CEA) and cost-consequence analysis (CCA) [24]. A CEA incorporates cost and a single measure of effectiveness into a metric represented by an incremental cost-effectiveness ratio (ICER) [25]. An ICER is calculated by dividing the difference in cost by the difference in outcome measure between 2 interventions, often treatment and control conditions in randomized controlled trials (RCTs). A CCA reports various costs and outcomes to enable decision makers to decide which are most relevant to their context and perspective [24]. It can capture costs to the user, system, and society and portray health, nonhealth, and nonpatient impacts [24].

An economic evaluation will be performed alongside the Better Nights, Better Days for Children with Neurodevelopmental Disorders (BNBD-NDD) RCT (ClinicalTrial.gov ID NCT02694003), which seeks to determine the efficacy of the eHealth behavioral intervention compared to usual care. BNBD-NDD is a parent-implemented eHealth behavioral program delivered to parents to enable accessible and effective care for insomnia in children with NDDs. The web-based nature of BNBD-NDD allows it to be readily scalable to reach many users. BNBD-NDD is transdiagnostic because it targets children ages 4 to 12 years with diagnoses of mildly to moderately severe attention-deficit/hyperactivity disorder (ADHD), autism spectrum disorder (ASD), cerebral palsy (CP), and fetal alcohol spectrum disorder (FASD). Although these 4 conditions present a broad range of cognitive, physical, and social characteristics, they are all associated with higher than normal rates of insomnia [26,27].

This protocol describes the BNBD-NDD economic evaluation, consisting of a CEA to evaluate the cost-effectiveness of BNBD-NDD in children with NDD compared with usual care and a CCA to examine outcomes beyond QoL. A CEA will be undertaken using the parent-reported Pediatric Quality of Life Inventory (PedsQL) questionnaire total scores, which will assess the children’s QoL. The QoL measure is used in economic evaluation because it allows comparison of the health outcomes of competing alternative interventions over time and across populations [28]. In addition, to illustrate a more comprehensive picture of BNBD-NDD’s impacts, a CCA will also be conducted to examine outcomes beyond the children’s health-related quality of life (HRQoL), including children’s sleep, parents’ daytime functioning, and psychosocial health.

Understanding the added value of BNBD-NDD is essential to informing the intervention's scale and spread. Thus, this protocol aims to complement the BNBD-NDD RCT and describe the economic evaluation from both family and societal perspectives.

## Methods

### Recruitment

Participants were recruited for the BNBD-NDD trial nationwide in Canada and stratified based on the parent-reported primary NDD diagnosis of their child. Potential parent participants were targeted via three groups: (1) parents of children with NDDs, (2) external stakeholders and organizations, and (3) the media. Internet-based tools were primarily used for recruitment, notably this study’s website and social media, as well as print materials and presentations to health care professionals.

### Inclusion Criteria

Participants must (1) reside in Canada and (2) be caregivers of children (3) ages 4 to 12 years who have been diagnosed with (4) ADHD, ASD, CP, or FASD by a health care provider and (5) meet the research criteria for insomnia. Insomnia criteria (ie, sleep onset disturbance) were characterized by at least 2 of 3 symptoms (>20 minutes to fall asleep, parent remaining in room for sleep onset, and more than 2 reunions) occurring 2 to 4 times per week for at least a month [29]. Additionally, these caregivers must have (6) regular access to a high-speed internet connection, (7) an email account, and (8) be proficient in English for everyday tasks.

### Sample Size Calculation

The BNBD-NDD RCT was powered to detect a group by time interaction across the 3 data collection time points (baseline, 4-month, and 8-month) and between the BNBD-NDD and usual care groups. The primary sleep outcome was the Disorders of Initiating and Maintaining Sleep (DIMS) [22] variable. To achieve a power of 0.80 with an α of .05, 39 participants per group would have to complete the DIMS measure at 8 months. Based on this analysis and the anticipated postenrollment dropout rate of 50% from a previous BNBD RCT, an estimated 160 participants were targeted for enrollment at baseline.

### Randomization and Blinding

Participants were randomized using a block stratified approach with 1:1 allocation to either the intervention (BNBD-NDD) or the usual care group and were not blinded to their assigned group. Stratification was by parent-reported primary NDD diagnosis.

### Study Groups

Participants were randomized to either the intervention group or the usual care group. Both groups may use alternative resources and other programs or services during this study. The intervention group was provided access to the BNBD-NDD program for 8 months after randomization (the duration of this study). As such, participants in the intervention group were regarded as receiving BNBD-NDD plus usual care. The usual care group could access BNBD-NDD after completing all follow-up assessments at 8 months.

### BNBD-NDD Intervention

BNBD-NDD is an eHealth program for parents or caregivers of children with NDDs and insomnia. The program is parent-implemented, incorporating transdiagnostic, evidence-based behavioral approaches, such as sleep education, stimulus fading, and scheduled awakenings, and presents NDD-specific strategies. The intervention is accessible via desktops, laptops, tablets, and mobile devices.

BNBD-NDD includes 5 sessions for participants to work through sequentially, with the recommended completion time ranging from 5 to 10 weeks. The sessions cover the following topics: (1) about BNBD-NDD; (2) healthy sleep practices; (3) settling to sleep independently; (4) night waking, napping, and early morning awakenings; and (5) looking back and ahead. Participants may access and complete the program at times that are convenient for them. Treatment fidelity and adherence were evaluated through process measurements and user statistics gathered by the intervention platform software. Participants may withdraw from the program at any time and are invited to provide reasons for withdrawal through open-ended questions.

### Perspective

Costs and outcomes are weighed differently by various decision makers. A way to determine the most pertinent perspective is by asking whom the analysis is intended to inform [30]. As BNBD-NDD is designed as a direct-to-user product, with the program’s users and beneficiaries being the parents and their children with NDDs and insomnia, this economic evaluation aims to inform them of the costs and effects of the program. The reference case costing will be completed from the perspective of the family payer (in this case, the parents). In accordance with recommendations from the Canadian Agency for Drugs and Technologies in Health, the reference case analysis will evaluate the QoL outcome for children, the primary target beneficiaries of the program [25]. In the nonreference case analysis from a societal perspective, the outcomes for caregivers (ie, daytime fatigue, psychosocial health), specific measures of children's sleep (ie, DIMS score), and societal costs (ie, treatment utilization [TU]) will also be examined.

### Time Horizon

In the BNBD-NDD RCT, data collection occurred at baseline, 4 months, and 8 months. The reference case CEA and secondary CCA will use an 8-month time horizon, focusing on baseline and 8-month data; 8-month data are used to best capture the longer term costs and effects.

### Outcomes for Economic Evaluation

PedsQL is an instrument that measures generic (rather than disease-specific) HRQoL and has been shown to have appropriate psychometric properties when used with children with NDDs [31]. It can be used to evaluate HRQoL in healthy children, youths, and those with medical conditions [32]. PedsQL uses a modular approach with 4 multidimensional scales on physical, emotional, social, and school functioning [33]. Parents rate each item using a 5-point Likert scale from 0 (never) to 4 (always) [31]. A Total Scale Score is calculated by adding all of the items and dividing the total by the number of items answered in all scales [33]. Mean Scale Scores are computed by dividing the sum of the items by the number of items answered in the scale [33]. Depending on the age of the participant's child, 3 parent-report versions were used (for ages 2-4 years, 5-7 years, and 8-12 years) to accommodate the age range of this study sample. The difference between the intervention and usual care groups’ average Total Scale Score in parent-proxy reports of their children’s PedsQL at baseline and 8 months will be evaluated in the reference case CEA. Secondary analyses will examine each of the 4 modules and age group trends; it is noted that inconsistent reporting of the school functioning section may occur due to age and the time of year of data collection (off-school period). The scoring and missing data (where applicable) will be completed in accordance with the PedsQL user manual [34].

In the CCA, the children’s sleep and parental outcomes are analyzed by comparing baseline and 8-month data and looking at changes in sample averages. Based on parent-reported questionnaire data, the DIMS score quantifies the severity of children's sleeping problems. The Single Item Fatigue Impact Scale [35] and the Depression, Anxiety, and Stress Scales [36] are used to evaluate the daytime fatigue and psychosocial health, respectively, of parents. Single Item Fatigue Impact Scale is adapted from the 40-item Fatigue Impact Scale [35]. The Depression, Anxiety, and Stress Scales have demonstrated adequate construct validity and high reliability [36].

### Resource Use and Costing

#### BNBD-NDD Cost

The direct cost of BNBD-NDD to the families is primarily the retail cost of the intervention. As the program was offered free of charge for our RCT, we used the average of parents’ (proxy payers for their children) reported willingness to pay. The participating families were expected to have internet access, which was not assessed or included in the direct costs. Retail pricing will be explored further from the willingness to pay data, which obtained a hypothetical willingness to pay (HWTP) by directly asking for the maximum cost participants would pay for the program. The HWTP responses may contain hypothetical bias influenced by factors such as the absence of a budget constraint effect and strategic answering, as some may perceive their input to potentially affect future pricing [37].

#### Treatment Utilization

The TU questionnaire [38] estimated the health care services families sought to address their children’s sleep problems, including publicly and privately funded ones. Care may be provided by family physicians, physician specialists (eg, pediatricians), and other health professionals (eg, psychologists). The mean frequency and costs of these services at baseline and at 8 months for the BNBD-NDD and usual care groups will be compared to see if BNBD-NDD impacted the usage rate. Out-of-pocket expenses reported for a health professional’s or provider’s help will also be assessed. Unit costs will be estimated using publicly available resources, such as the Canadian Agency for Drugs and Technologies in Health [39].

### Data Analysis

#### Overview

For the reference case CEA analysis, the mean sample PedsQL at baseline and 8 months will be compared. Regression analyses will examine the impacts of baseline characteristics (geographical region, age groups, sex, household income, and NDD diagnosis) on the efficacy measures. For all regression analyses, the mean difference between groups will be reported, as well as the variance, *P* value, and goodness of fit using *R*^2^.

#### Reference Case CEA

In the reference case analysis using the family payer perspective, the ratio of the difference in mean cost between groups to the difference in mean PedsQL total score per group will be used to estimate the ICER. An ICER will be calculated from the family’s perspective.

ICER = Costs_(BNBD-NDD + usual care)_ − Costs_(usual care)_ / Effect_(BNBD-NDD + usual care)_ − Effect_(usual care)_

#### Secondary CCA

The CCA will assess the impacts on children’s sleep disturbance severity, parents’ daytime functioning, and psychosocial health. It adopts a broader perspective, providing a wider range of relevant costs and outcomes [24]. This allows various decision makers to focus on aspects and contexts specific to them [24]. The costs examined will extend beyond the family and include the costs of reported publicly and privately funded services sought.

#### Subgroup Analysis

Subgroup analyses will be performed to determine if there are any trends, similarities, or differences in the mean outcomes of the 4 NDD diagnostic groups (ADHD, ASD, CP, and FASD); the CP and FASD groups are likely to have relatively fewer participants than the ADHD and ASD groups due to their lower prevalence.

#### Sensitivity Analysis

Sensitivity analysis will test variations in assumptions regarding estimated costs and reported health care usage. With the retail cost of the intervention under discussion, a range of prices will be explored based on the HWTP data, and the corresponding ICERs will be calculated. Parameter uncertainties associated with TU will be explored through scenario analyses, incorporating the 95% CI of mean estimates.

### Missing Data

Multiple imputation techniques are used to address missing data. This method works well for longitudinal data and is robust to violations of the nonnormality of the variables analyzed. Participants who do not complete both the baseline and the 8-month follow-up will not be included in the analysis. Where applicable, the measures’ user guides on missing data will be followed. For the PedsQL, the means of completed items in the scale will be imputed when 50% or more of the items are completed [34].

### Discount Rate

As all costs and outcomes assessed are within 1 year, a discount rate will not be applied [25].

### Ethics Approval

All study participants provided informed consent to participate in the BNBD-NDD RCT. Ethical approval for the BNBD-NDD trial (REB-1020681) was granted by the IWK Health Centre Research Ethics Board and the associated economic evaluation by the IWK REB and the University of Calgary Conjoint Health Research Ethics Board (REB22-0694). This study complies with all applicable standards and procedures of the IWK REB, the Personal Health Information Act, the Personal Information International Disclosure Protection Act, and the Tri-Council Policy Statement: Ethical Conduct for Research Involving Humans.

## Results

The BNBD-NDD RCT data collection started in June 2019 and ended in April 2022. Research funding was obtained from the Kids Brain Health Network in 2015. Ethics were approved by the IWK Ethics Board and Conjoint Health Research Ethics Board in January 2019 and June 2022, respectively. The data examining the primary outcomes of this RCT are currently being analyzed. The relevant data for the economic evaluation have been cleaned and are being prepared for analysis.

## Discussion

In this paper, we have described an economic evaluation protocol for the BNBD-NDD eHealth intervention, which aims to improve insomnia in children with NDDs. The economic evaluation will identify, measure, and value the costs and outcomes of BNBD-NDD compared to usual care. The reference case CEA will assess the costs and QoL of the children, and the CCA will examine measures beyond QoL for the children and their caregivers.

The prevalence of sleep disorders has been observed to be higher in children with NDDs relative to TD children [40]. BNBD-NDD has the potential to deliver an evidence-based and cost-effective insomnia intervention to children with NDDs, which is critically important given the lack of available treatments. The eHealth delivery system may also overcome some existing barriers to treatment access. If cost-effective, this program can improve sleep outcomes and positively affect the well-being of children with NDDs and their caregivers. To our knowledge, BNBD-NDD is the first eHealth insomnia intervention for children with NDD. No published reports have evaluated internet-based interventions for insomnia in preschool- and school-aged children; 1 study has reported an effective internet intervention for sleep disturbances in infants and toddlers [41].

Some limitations of this study are the uncertainties with cost estimates, the parental-reported measures on behalf of the children, the recall bias of their children’s 12-month service usage, and the lack of objective outcome data. With family and societal payer perspectives, the breadth of the costing analysis is limited by the collected cost data in the RCT and thus likely to be an underestimation of real-world costs. While the CCA provides a wider scope of relevant outcomes and costs for decision makers, the interpretation of the results may be prone to subjective bias [24]. The BNBD intervention would benefit from real-world evidence to evaluate its effectiveness and also explore its applicability to children with other medical conditions.

Despite a direct-to-user approach for the intervention, the economic evaluation can stimulate conversations with relevant stakeholders, better understand the efficacy of web-based alternatives, and provide evidence to support potential public payer funding initiatives. Dialogue on the integration of eHealth interventions in the delivery of care in the health system is another area for future examination. The findings of this study will provide users and other stakeholders with a holistic picture of the costs and impacts of BNBD-NDD and play a role in informed decision-making for resource allocation.

## Abbreviations

ADHD: attention-deficit/hyperactivity disorder
ASD: autism spectrum disorder
BNBD: Better Nights Better Days
BNBD-NDD: Better Nights, Better Days for Children with Neurodevelopmental Disorders
CCA: cost-consequence analysis
CEA: cost-effectiveness analysis
CP: cerebral palsy
DIMS: Disorders of Initiating and Maintaining Sleep
FASD: fetal alcohol spectrum disorder
HRQoL: health-related quality of life
HWTP: hypothetical willingness to pay
ICER: incremental cost-effectiveness ratio
NDD: neurodevelopmental disorder
PedsQL: Pediatric Quality of Life Inventory
QoL: quality of life
RCT: randomized controlled trial
TD: typically developing
TU: treatment utilization

## Data Availability

The data sets generated and analyzed during this study are not publicly available as the participants of this study did not give written consent for their data to be shared publicly. Due to the sensitive nature of the research, supporting data are not available.
